# Giant secreting adrenal myelolipoma in a man: a case report

**DOI:** 10.1186/1752-1947-5-298

**Published:** 2011-07-09

**Authors:** Alfio Brogna, Giuseppe Scalisi, Rosario Ferrara, Anna M Bucceri

**Affiliations:** 1 Department of Internal Medicine, Gastroenterology Unit, S. Luigi Hospital, Viale Fleming 24, I-95100 Catania, Italy

## Abstract

**Introduction:**

Adrenal myelolipoma is a rare, benign neoplasm that is usually asymptomatic, unilateral and nonsecreting. It develops within the adrenal gland and is composed of mature adipose tissue with elements of the hematopoietic series. We describe the case of what is, to the best of our knowledge, one of the largest secreting adrenal myelolipomas reported in the literature.

**Case presentation:**

A 52-year-old Caucasian man of medium build who had had moderate hypertension for three years presented to our hospital. He had no other significant symptoms. His hypertension was pharmacologically treated. He came to our hospital to undergo abdominal ultrasonography during a clinical checkup. The ultrasound scan showed the presence of a voluminous hyperechoic mass interposed between the spleen and the left kidney. It was reported as a myelolipoma of the left kidney on the basis of its structural characteristics and position. Computed tomography confirmed our diagnosis. All preoperative biochemical tests were normal, with the exception of high serum cortisol, which was being overproduced by the lesion and was probably responsible for the patient's hypertension. He underwent successful surgery, and his postoperative course was uneventful. The pathologic examination of the lesion confirmed the diagnosis of adrenal myelolipoma. The patient's blood pressure returned to within the normal range.

**Conclusions:**

The "incidental" discovery of an adrenal mass requires careful diagnostic study to plan adequate therapeutic management. Both of the primary investigations at our disposal, ultrasound and blood tests (adrenal hormones), helped in rendering the diagnosis and allowed us to move toward the most appropriate treatment, taking into account the size of the tumor and its probable hormonal production.

## Introduction

The myelolipoma is a rare, benign neoplasm composed of mature adipocytes and hematopoietic tissue. It was first described by Gierke in 1905 and subsequently by Oberling in 1929, who used the term "myelolipoma" [1]. In the past, the finding of adrenal lesions was made possible by autopsy or by clinical presentation, related either to the massive growth of the gland or to altered hormone production. Today these tumors can be discovered incidentally because of the wide use of diagnostic imaging methods, such as ultrasonography (US), computed tomography (CT) and magnetic resonance imaging [2]. Although the incidence of these tumors is unknown, it is thought to be between 0.08% and 0.4%. Men and women seem to be equally affected by these tumors. Adrenal myelolipoma is most commonly found between the fifth and seventh decades of life [3]. In the latter age group, its low incidence seems to have increased by 0.2% to 10% since the mid-1990s [4].

These lesions are usually unilateral and asymptomatic. A certain number of bilateral tumors have been described in the literature. Myelolipomas are often smaller than 4 cm in diameter, although they can reach wider sizes [5]. The largest adrenal myelolipoma reported in the literature weighed 6 kg and measured 31 cm × 24.5 cm × 11.5 cm [6,7]. After surgical excision, these lesions generally do not recur. They are generally nonsecreting, although an overproduction of adrenal hormones is described in some cases. More than 25 cases of endocrine dysfunction associated with myelolipoma have been reported in the English- and Japanese-language literature [5,8,9]. Here we describe one of the largest cortisol-secreting myelolipomas ever reported in the literature [10,11].

## Case presentation

**Figure 1:**
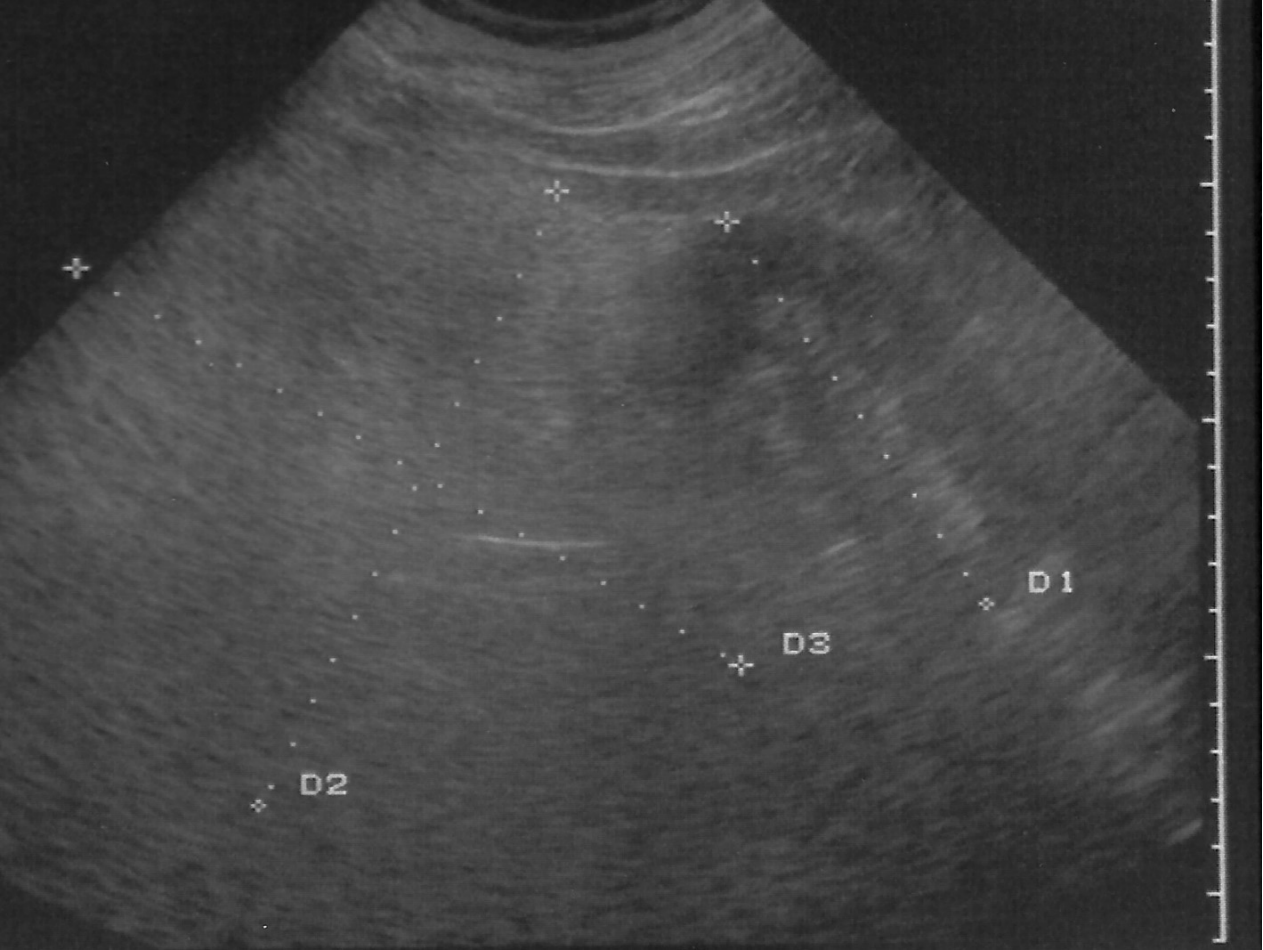
**US scan of the abdomen showing a myelolipoma**. A homogeneous hyperechoic mass in the upper abdomen that displaces the left kidney downward is shown.

**Figure 2:**
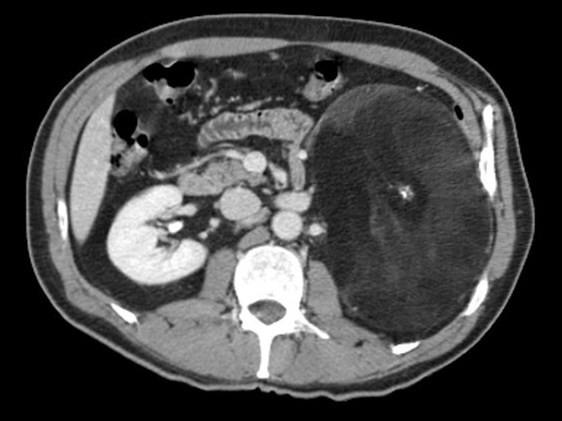
**Myelolipoma identified by CT**. Contrast-enhanced CT scan of the upper abdomen shows a large heterogeneous mass covering the upper left retroperitoneal space.

We report the case of a 52-year-old Caucasian man of medium build who had had moderate hypertension for three years. He referred to no other noticeable symptom. His hypertension was pharmacologically treated. The patient came to our hospital to undergo abdominal ultrasonography during a clinical checkup. US showed the presence of a large hyperechoic mass (Figure 1) with non-well-defined boundaries that made it difficult to measure its exact size. It seemed to be 20 cm in diameter. The mass was interposed between the spleen and the left kidney. The left kidney was displaced downward. A probable myelolipoma of the left adrenal gland was diagnosed. Contrast-enhanced CT was proposed to confirm the diagnosis. The abdominal CT scan confirmed the presence of an expansive lesion (Figure 2) largely occupying the left hemiabdomen. Such a lesion rising from the left adrenal gland had frankly fat content, with delta numbers equal to a mean of -130 HU. It had sharp, regular boundaries with starlike central calcifications. It was suggestive of a giant left adrenal myelolipoma. The spleen was pushed against the costal wall and slightly displaced cranially, while the left kidney was pushed downward and the left renal artery and vein were stretched. The compression exerted on the vein caused a dramatic ipsilateral spermatic varicocele. The pancreatic gland at the tail, the splenic artery and vein as well as the left colonic flexure were displaced anteriorly. Preoperatively, his routine biochemistry was normal (hemochrome with formula, liver and kidney function), while his serum cortisol level was increased (cortisol at 10 a.m. = 743 nmol/L, cortisol at 2 p.m. = 637 nmol/L, cortisol at 6 p.m. = 649 nmol/L; ranges, 7 a.m. to 10 a.m., 171 to 536 nmol/L; 4 to 8 p.m., 64 to 327 nmol/L). His glucose level was 122 mg/dL (range, 60 to 110 mg/dL). His urinary catecholamines were normal.

A surgical left adrenalectomy was performed. The surgically removed myelolipoma showed it to have an oval shape (about 25 cm × 20 cm × 20 cm), and it weighed 4.4 kg. Sectioning revealed areas representing well-differentiated fat. The histological examination revealed the presence of adipose tissue with hematopoietic elements without signs of cellular atypia, thus confirming the initial diagnosis. The patient's postoperative course was altogether uneventful.

## Discussion

Adrenal myelolipoma is often an "incidentaloma," since its diagnosis is frequently based on autopsic findings or upon diagnostic imaging examinations performed for reasons unrelated to its presence. These tumors are rare, although they are increasingly being detected because of wider use of diagnostic imaging techniques. They are benign and nonfunctional tumors composed of mature adipocytes and active hematopoietic elements. Their histogenesis is uncertain. According to some authors, the tumor develops from residual embryonic cells of the bone marrow that, after embolization, reach the adrenal gland. According to a recent theory, myelolipoma originates from a metaplastic process of the reticuloendothelial cells within adrenal capillaries. Some authors even consider the development of adrenal myelolipomas as a response to various endocrine stimulations [2]. This hypothesis is confirmed by the autopsic findings of myelolipomas in patients who died as a result of chronic systemic diseases. Other contemporary authors have speculated about a stressful lifestyle and an unbalanced diet as factors that may be involved in the pathogenesis of this tumor [4]. An increase in mass is very slow, becoming symptomatic only when, because of an increase in the volume of tumor compression, phenomena are diagnosed and/or the tumor spreads to neighboring organs. Hemorrhage or necrosis that occur within the tumor can cause pain. Hypertension and hematuria may be other symptoms reported by the patient, who usually has a strong constitution or may be obese [2]. Myelolipomas associated with Cushing's syndrome, Conn syndrome and congenital adrenal hyperplasia caused by a deficiency of 21-α hydroxylase or 17-α hydroxylase [3,10] have been described. An unusual and unexplained observation is the predominance of the tumor borne by the right adrenal gland [4]. The differential diagnosis should include renal angiomyolipoma, retroperitoneal lipoma and liposarcoma [12].

In the clinical case we have described here, it is interesting to note the hyperincretion of cortisol by the myelolipoma. This hyperincretion was probably responsible for the patient's hypertension, since his blood pressure was normalized after surgical removal of the tumor and thereafter his blood pressure remained within normal values.

Another rare feature to consider is the left-sided position of the myelolipoma in our patient, because they have been reported in the literature to be prevalent mostly on the right side. This location has probably facilitated the rise of a left spermatic varicocele even if the patient is asymptomatic.

## Conclusions

The "incidental" discovery of an adrenal mass requires careful diagnostic study to plan an appropriate treatment. Imaging techniques at our disposal today can help the clinician to render the diagnosis. Since myelolipomas consist mainly of adipose tissue, their sharp hyperechogenicity observed on US may orient the clinician toward the diagnosis, but US does not yield any certain detail about nature of lipomatous lesions. CT can clarify the nature of incidentalomas, as in our patient, and can indicate the best treatment, taking into consideration the tumor size and its possible hormone production. Biochemical studies are also useful and necessary in the typing of the mass.

## Abbreviations

CT: computed tomography; MRI: magnetic resonance imaging; US: ultrasonography.

## Consent

Written informed consent was obtained from the patient for publication of this case report and any accompanying images. A copy of the written consent is available for review by the Editor-in-Chief of this journal.

## Competing interests

The authors declare that they have no competing interests.

## Authors' contributions

AMB and GS collected the data and drafted the manuscript. AB and RF took care of the patient during his hospitalization. All authors read and approved the final manuscript.
